# Defining Human Regulatory T Cells beyond FOXP3: The Need to Combine Phenotype with Function

**DOI:** 10.3390/cells13110941

**Published:** 2024-05-30

**Authors:** Chelsea Gootjes, Jaap Jan Zwaginga, Bart O. Roep, Tatjana Nikolic

**Affiliations:** Laboratory of Immunomodulation and Regenerative Cell Therapy, Department of Internal Medicine, Leiden University Medical Center, 2333 ZA Leiden, The Netherlands; j.j.zwaginga@lumc.nl (J.J.Z.); t.nikolic@lumc.nl (T.N.)

**Keywords:** Tregs, human, FOXP3, clones, function, type 1 diabetes, autoimmune disease

## Abstract

Regulatory T cells (Tregs) are essential to maintain immune homeostasis by promoting self-tolerance. Reduced Treg numbers or functionality can lead to a loss of tolerance, increasing the risk of developing autoimmune diseases. An overwhelming variety of human Tregs has been described, based on either specific phenotype, tissue compartment, or pathological condition, yet the bulk of the literature only addresses CD25-positive and CD127-negative cells, coined by naturally occurring Tregs (nTregs), most of which express the transcription factor Forkhead box protein 3 (FOXP3). While the discovery of FOXP3 was seminal to understanding the origin and biology of nTregs, there is evidence in humans that not all T cells expressing FOXP3 are regulatory, and that not all Tregs express FOXP3. Namely, the activation of human T cells induces the transient expression of FOXP3, irrespective of whether they are regulatory or inflammatory effectors, while some induced T cells that may be broadly defined as Tregs (e.g., Tr1 cells) typically lack demethylation and do not express FOXP3. Furthermore, it is unknown whether and how many nTregs exist without FOXP3 expression. Several other candidate regulatory molecules, such as GITR, Lag-3, GARP, GPA33, Helios, and Neuropilin, have been identified but subsequently discarded as Treg-specific markers. Multiparametric analyses have uncovered a plethora of Treg phenotypes, and neither single markers nor combinations thereof can define all and only Tregs. To date, only the functional capacity to inhibit immune responses defines a Treg and distinguishes Tregs from inflammatory T cells (Teffs) in humans. This review revisits current knowledge of the Treg universe with respect to their heterogeneity in phenotype and function. We propose that it is unavoidable to characterize human Tregs by their phenotype in combination with their function, since phenotype alone does not unambiguously define Tregs. There is an unmet need to align the expression of specific markers or combinations thereof with a particular suppressive function to coin functional Treg entities and categorize Treg diversity.

## 1. Introduction

It is important for the immune system to have the ability to control inflammatory responses and prevent chronic reactions against self-antigens. In addition, foreign antigens introduced by pregnancy, organ transplants, or commensal bacteria in the intestine also need to be tolerated by the immune system. One of the mechanisms to control immune responses involves regulatory T cells (Tregs). Tregs reside in lymphoid and non-lymphoid organs and adapt to their environment in order to maintain tissue homeostasis. An imbalance between inflammatory T cells (i.e., effector T cells or Teff) and Tregs can cause a loss of tolerance and disease. For example, decreased Treg counts or low suppression activity are associated with autoimmunity [1] and chronic inflammation [2]. Persistent Treg activity, on the other hand, can favor tumor progression [3], chronic infections [4], and low responsiveness to vaccinations [5,6].

An overwhelming variety of Tregs has been described, either generated in vitro or isolated from human tissues in various pathological situations, using novel phenotypic markers and different functional assays [7,8,9,10,11,12,13]. This complicates the assessment of whether a certain Treg is related to a previously described Treg type and reaching a conclusion as to whether a disbalance between the same Treg and Teff subsets supports different pathologies. One of the efforts to characterize Tregs was made by establishing a minimum level of information on in vitro cultured Tregs [14], but a consensus on how to characterize human Tregs ex vivo is lacking at the moment. With different, partially overlapping phenotypic marker combinations, and with a universal marker for Tregs lacking, only the functional capacity to inhibit immune responses defines all Tregs and distinguishes these from Teff cells. An additional limitation of characterizing immune cells by the available surface markers is that these markers often do not correlate with the functional diversity of Tregs. In this review, we revisit general Treg knowledge, beliefs, and simplifications since some of these are still used as a standard to identify human Tregs. We propose to combine the phenotype with suppressive functional features to characterize the population of interest and define phenotype markers that align with particular suppressive functionalities to classify Treg populations and understand their relations.

## 2. Revisiting Human Treg Phenotypes

A ubiquitously accepted definition of Tregs was of any cell expressing FOXP3, but this proved problematic for human Tregs. In search of an alternative, improved techniques such as multidimensional flow cytometry and single-cell sequencing revealed many different Treg subtypes, introduced new markers that were claimed to be Treg-specific, described multiple Treg origins and antigen specificities, and challenged us to revisit the omnipresent view of the identity of human Treg cells, Treg phenotypes, and functions, as well as better delineation from Teff cells.

### 2.1. FOXP3 Is Neither Specific nor Exclusive to Human Tregs

The transcription factor Forkhead box protein 3 (FOXP3) was discovered in scurfy mice [15]. These mice have a missense mutation in the *Foxp3* gene and, therefore, lack Foxp3 expression in T cells, resulting in a lymphoproliferative disease with multiorgan inflammation [16,17,18]. While, in humans, autoimmunity can occur independently of dysfunctional Tregs [19,20,21,22], patients with IPEX (immune dysfunction/polyendocrinopathy/enteropathy/X-linked) syndrome often show mutations in the *FOXP3* gene [23,24,25,26,27,28] and present with symptoms like severe enteropathy, polyendocrinopathy, and immune dysregulation, similar to scurfy mice [29,30,31]. However, not all IPEX patients have a mutation in *FOXP3*, implying that immune (dys)regulation can occur either downstream or independently of FOXP3 [32,33,34,35,36]. Indeed, a study involving 15 IPEX patients showed that Treg signature genes were expressed despite a loss-of-function mutation in *FOXP3*, indicating that Tregs can exist without functional FOXP3 [37,38]. In vitro-induced or expanded antigen-specific Tregs can be negative or can transiently express FOXP3 [7,39,40,41,42,43,44]; therefore, FOXP3 has been primarily associated with the so-called naturally occurring Tregs (nTregs) rather than acting as a universal Treg marker.

Furthermore, in human T cells, FOXP3 is expressed in non-suppressive Teff cells shortly after T cell receptor (TCR) stimulation (e.g., with anti-CD3) [45,46,47] (Table 1). Hence, detecting FOXP3 in T cells alone is not sufficient to specify Tregs in cross-sectional analyses of human tissue samples, since staining for FOXP3 in inflammatory lesions may include recently activated Teff cells while leaving some induced Tregs with unstable FOXP3 that are undetected and ignored. On the other hand, analyzing the Treg-specific demethylated region (TSDR) can discriminate between Tregs with sustained FOXP3 expression and Teff transiently expressing FOXP3 [48]. This method, however, is not unambiguous since the TSDR can be partially demethylated in bonafide Tregs [49], as well as in some Teff subpopulations [50].

Notwithstanding the importance of FOXP3 as a checkpoint in human Treg differentiation and the suppression of Teff, and considering the available literature, we conclude that FOXP3 in humans does not exclusively identify Tregs, or all Tregs, and should not be used as a stand-alone proxy for immune regulation in vivo [51].

### 2.2. The Complexity of the Treg Universe, as Revealed by Their Phenotypes

The appreciation of the limitations of FOXP3 in humans as an unambiguous Treg marker has stimulated an extensive search for other molecules that differentiate suppressive from non-suppressive T cells. This approach, however, made defining Tregs in human samples even more difficult since it unraveled a whole universe of Treg subsets, based on either specific phenotypes, tissue compartments, or functions. 

For classification, human Tregs have been divided into three groups: tTregs, generated in the thymus and also commonly known as nTregs, pTregs, generated in the peripheral tissues, and iTregs, induced in vitro. The tTregs/nTregs express CD25 and FOXP3 and lack CD127. They can sometimes express molecules like cytotoxic T-lymphocyte-associated protein 4 (CTLA4), glucocorticoid-induced TNFR-related protein (GITR), latency-associated peptide (LAP), and/or lymphocyte-activation gene 3 (Lag-3), and can produce inhibitory cytokines such as interleukine-10 (IL-10), transforming growth factor β (TGF-β), and/or IL-35. However, not all the proposed molecules are expressed on all nTregs, and over twenty subsets have been described within the nTregs population [52]. It remains a subject of debate how to distinguish pTregs from tTregs/nTregs (markers like Helios and Neuropilin (Nrp-1) have been proposed but discarded since the publication of [53,54,55,56]) and whether some of the pTregs found in patients compare to those induced in vitro, such as Type 1 regulatory T cells (Tr1), which are characterized by CD25^lo^, FOXP3^neg^, Lag-3^pos^, and CD49b^pos^ expression, and the production of high levels of IL-10 [7,39,40,41,42], along with Type 2 regulatory T cells (Tr2 or Th3), which produce high levels of TGF-β and express CD25^lo^, FOXP3^neg^, LAP^pos^, CD69^pos^, CTLA4^lo^, and the GITR^lo^ phenotype [43,44].

**Figure 1:**
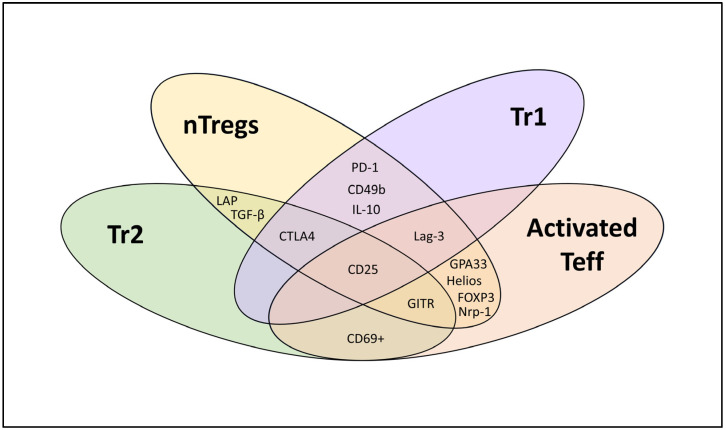
Phenotypic characterization of different T cell subsets (nTregs, Tr1, Tr2, and activated effector T cells (Teff)).

The continuous splitting of Treg populations into subsets on the basis of new candidate markers adds to the confusion when classifying Treg sub-entities, which can share phenotypic and functional features irrespective of their origin that sometimes overlap with activated Teff cells (Figure 1). Furthermore, supposed Treg markers such as GITR, Lag-3, and glycoprotein A33 (GPA33) and others (Table 1) can be expressed by activated Teff cells to regulate/modulate T cell activation and may be linked to the contraction of T cells after activation to regain immune homeostasis [57,58,59,60,61]. For example, Lag-3 is upregulated on all T cells upon TCR activation or by certain cytokines [57]. By binding to HLA class II, Lag-3 inhibits the early steps of the TCR pathway, suppressing any subsequent stimulation [62]. Activated T cells (Tregs and Teff cells) thus express Lag-3, enabling them to blunt any overt immune reactivity and contract Teff expansion. Nevertheless, when Tregs express Lag-3, they do so with greater magnitude compared to CD4+ Teff cells [63], giving them an advantage when interacting with and inhibiting antigen-presenting cells (APCs). Thus, some of the described markers may not be Treg-specific but are, nevertheless, employed by these cells for their regulatory function. These markers should be monitored even though their changing expression rates, upon activation, hamper the qualification of Treg subtypes or distinction between Tregs and Teff cells.

When cytokines, chemokines, and other transcription factors are included, further subsets of Tregs have been suggested, such as IL-35-producing induced Tregs (iTr35, FOXP3^−^IL-35^+^) [64] or TGFb and IL-10-producing Tregs induced by B cells (Tregs-of-B cells, FOXP3^−^ IL-10^+^ TGF-β^+^) [65,66,67,68] and the regulatory subtype of Tfh cells (Tfr, FOXP3^+^ CXCR5^+^ CD25^+/−^ Bcl-6^+^ PD-1^+^ ICOS^+^) [69,70]. Adding to the confusion, the newly discovered Treg subtypes have been given new names without testing their similarity to previously described subsets (indeed, we, too, are guilty as charged; see [49]), resulting in many seemingly different but potentially related Treg subtypes, making the comparison of Tregs between studies difficult. 

**Table 1:** Markers claimed to be specific for regulatory T cells (Tregs) but shown to also be expressed by activated effector T cells (Teff).

Markers	‘Treg-Specific’ [Reference]	Also Expressed in Teff Cells [Reference]
4-1BB/CD137	[71]	[72]
CD25	[8]	[46,73]
FOXP3	[74,75,76]	[45,46,47]
GARP	[77]	[78,79]
GITR	[9,80]	[81]
GPA33	[82]	[61]
Helios	[54,83,84,85]	[53]
Lag-3	[86,87]	[88]
LAP	[10]	[79]
Neuropilin	[11,56]	[55]
OX40	[71]	[89]
TNFR2/CD120b	[90]	[91]

The challenge to measure all described Treg markers simultaneously, due to their intracellular expression or release and limited technical capacities, was partially addressed by multidimensional analyses (e.g., CyTOF analysis and spectral flow cytometry), revealing many distinct subpopulations of nTregs alone [52], and confirming the complexity of the Treg universe. These subsets may also represent nTregs in different development/activation stages, reflect their changes in time, their different tissues of origin, or variations in the functions they exert. Moreover, multidimensional phenotyping does not yet resolve the problem of discriminating between Tregs and Teff cells effectively [92]. In conclusion, the search for a universal Treg-specific marker, or one at least shared between most Treg subtypes but not with activated Teff cells, has not been successful as yet, and functional assays remain indispensable to characterize Tregs in concert with their phenotypes.

### 2.3. Does the Priming Site Determine the Treg Type?

In our view, the attempt to classify human Tregs based on their origin (i.e., where they acquired their regulatory function) into tTregs, pTregs, and iTregs does not reduce confusion since all T cells derive from the thymus by definition. While the priming and differentiation site of tTregs or pTregs/iTregs may differ, similarities in specificity, phenotype, or function cannot be excluded. The assumed differentiation of Tregs from thymocytes follows a similar route to that of conventional T cells, both requiring TCR-dependent signaling after engagement with human leukocyte antigen (HLA) class II molecules on specialized APCs presenting a variety of peptides derived mainly from available autoantigens [93,94,95,96,97]. The differentiation of nTregs is presumed to occur during negative selection, where high-avidity TCR/HLA-interactive T cells undergo apoptosis [98,99], low-avidity TCR/HLA interactive T cells favor egressing into conventional naïve T cells, and intermediate-avidity TCR/HLA interactive T cells would favor sustained FOXP3 expression and differentiate into nTregs/tTregs [100,101,102,103]. The low-avidity TCR/HLA-binding naïve T cells may subsequently develop into pTregs/iTregs upon encountering the antigens presented by tolerance-inducing APCs in specialized tolerogenic niches in the periphery or in vitro [49,92,104,105,106,107,108]. As a result, tTregs are believed to be biased toward the immune recognition of self-antigens presented in the thymus, whereas pTregs/iTreg cells will recognize non-self- and neoantigens not presented in the thymus, such as allergens, food, tumor- or stress-induced antigens, and microbiota [109,110]. This strict division, however, knows exceptions; we showed that Tregs reacting against the self-derived proinsulin peptide can be induced from naïve CD4 T cells by tolerogenic dendritic cells (tolDCs) [49,92], and these Tregs would be classified as iTregs. Also, IL-10-producing islet-specific CD4 T cells that qualify as pTregs were isolated from nondiabetic individuals ex vivo [111]. Thus, pTregs and iTregs can respond to self-antigens and are not exclusively biased toward recognizing non-self-antigens. 

## 3. Lessons from Treg Clones

Most studies of Tregs and Treg subtypes have been performed on polyclonal populations. Tregs in polyclonal populations can now be analyzed on a single-cell level [112,113,114]. However, to accurately investigate Treg diversity and relate phenotypes with functions, Tregs must be studied at the clonal level. Isolating and expanding such antigen-specific Treg clones, however, is extremely challenging since Tregs are endorsed with (self-) regulatory properties that impair their expansion. Nonetheless, attempts to propagate and characterize a range of a-specific expanded (e.g., with anti-CD3) or melanoma antigen family A3 (MAGE-A3)-specific Treg clones [115,116], or induced/expanded proinsulin-specific Treg clones did succeed [49,111]. Some MAGE-specific Tregs that were isolated from melanoma patients vaccinated with MAGE were CD4+CD25+ and demethylated FOXP3, whereas other CD4+ clones lacking CD25 could also suppress Teff cells, despite FOXP3 being methylated [116]. Autoreactive Tregs that were induced by tolDC against proinsulin were functionally and phenotypically indistinguishable from islet autoantigen-specific Tregs sorted directly from blood by IL-10 capture assays [49,111]. Unsupervised clustering of these proinsulin-specific Treg clones based on their regulatory features (including their function and phenotype, such as the intracellular expression of IL-10, IFN-γ, granzyme B, the surface expression of CTLA-4 and IL-10, IFN-γ, TNF, and IL-13 production in combination with functional characterization, including monocyte killing, the prevention of the activation of naïve T cells (the ‘classic’ suppression assay), and the inhibition of pre-primed, activated effector Th1 cells) revealed three discrete clusters, in spite of an identical induction protocol and the antigen-specificity of these Treg clones (Table 2) [49]. Cluster 1 involved Treg clones producing high amounts of cytokines (IL-10, TNF, IFN-γ, and IL-13), which were able to kill monocytes and inhibited both naïve T cells and, to a lesser degree, activated Th1 responses. Cluster 2 exhibited strong monocyte killing performance, often accompanied by granzyme B secretion, whereas cluster 3 defined efficient monocyte killers that also strongly inhibited both naïve T cell and Th1 responses. 

Clones from cluster 3 can induce infectious tolerance [117]. These iTregs, primed by tolDCs, were able to functionally modify proinflammatory mDCs to become anti-inflammatory dendritic cells (DCs) through mechanisms including ICOS-L and B7-H3 ligation. These converted DCs in turn lost their capacity to stimulate Teff cells and, instead, induced IL-10-producing Tregs from the naïve T cell repertoire. 

Surprisingly, the Treg clones from cluster 1 exhibited a completely inversed polarity of their TCR, leading to reversed docking onto the proinsulin peptide/HLA complex [49]; the α-chain and the β-chain of these TCRs interacted with the α-chain and β-chain of the HLA class II molecule, respectively. Nevertheless, this unorthodox TCR–peptide–HLA interaction resulted in HLA-restricted and antigen-specific (proinsulin) Treg activation. Thus, we propose that some Tregs may employ uniquely reversed TCR docking, contributing to their regulatory nature, a feature that would distinguish Tregs from Teffs. Indeed, similar inversed TCR docking has since been observed in the T cells of mice unresponsive to flu vaccination [118]. It is, therefore, important to consider that such a reverse TCR docking feature might be characteristic of antigen-specific Tregs when using TCRs in CAR or ‘avatar’ Treg models. Hence, endorsing Tregs with TCR derived from Teff may not necessarily result in immune regulation being exerted through the adopted TCR. 

In conclusion, Treg clones have taught us that even when Tregs share antigen-specificity, their phenotypic and functional characteristics divide them into clusters, supporting the finding that not all Tregs are equal and that functional features assigned to polyclonal populations may, in fact, represent combinations of separate mechanistic features asserted by different Treg clones. 

## 4. Functional Diversity of Tregs

**Figure 2:**
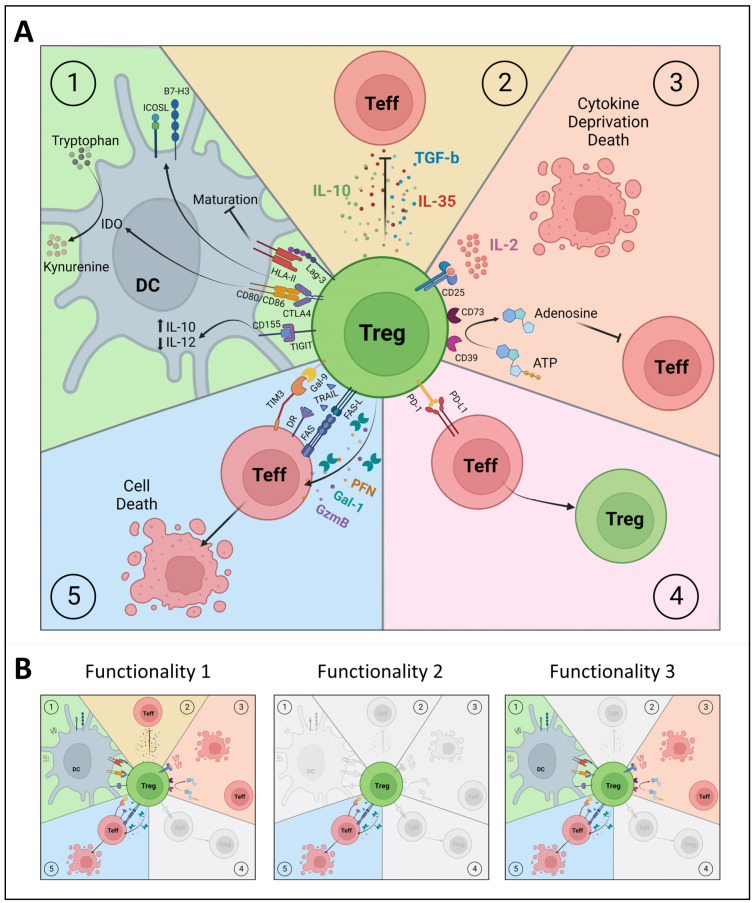
Functional characterization of polyclonal and clonal Treg subsets. (**A**) Different mechanisms of immunosuppression by regulatory T cells (Tregs): 1. Interaction with antigen-presenting cells (dendritic cell, DC). 2. Secretion of inhibitory or immunosuppressive cytokines. 3. Metabolic disruption. 4. Direct contact with effector T cells (Teff). 5. Induction of apoptosis. (**B**) The revisited Treg universe, based on functional properties at the clonal level rather than on phenotype at the polyclonal level. There are Tregs with a single inhibitory function (e.g., killing, Functionality 2), while other Tregs may exert multiple immunoregulatory functionalities (some functions might be shared, as with Functionalities 1 and 3). CTLA4: cytotoxic T-lymphocyte associated protein 4, DR: death receptor, Gal-1: galectin 1, Gal-9: galectin 9, GzmB: granzyme B, HLA-II: human leukocyte antigen class II, ICOSL: inducible co-stimulator ligand, IDO: indoleamine 2,3 dioxygenase, Lag-3: lymphocyte-activation gene 3, PD-1: programmed cell death protein 1, PD-L1: programmed death-ligand 1, PFN: perforin, TIGIT: T cell immunoreceptor with immunoglobulin and ITIM domain, TIM3: T cell immunoglobulin and mucin domain 3, TRAIL: tumor necrosis factor-related apoptosis-inducing ligand. Figures were created with Biorender.com.

Similar to our findings using the proinsulin-specific iTreg clones [49], nTregs may exert a range of regulatory mechanisms (reviewed in [119]), such as their interaction with and modulation of APCs, the release of inhibitory cytokines, interference with metabolic signals, direct interaction with Teff cells, and the induction of apoptosis (Figure 2). However, it remains unresolved whether all these mechanisms are employed simultaneously, or whether individual nTregs show different patterns of functionality. Since our clonal iTregs showed that clones within different phenotype clusters can share regulatory mechanisms (e.g., the induction of apoptosis), we propose that the detailed characterization of diverse regulatory mechanisms is indispensable for distinguishing different types of Tregs and should be included in Treg phenotyping. In the following section, we will briefly review various mechanisms of T cell regulation that should be considered when assessing Treg functionality (Figure 2).

### 4.1. Interaction with Antigen-Presenting Cells

There are several mechanisms by which Tregs interact with APCs; these can alter the maturation and/or function of DCs, affecting antigen presentation and T cell activation (Figure 2). DCs provide the required costimulation for Teff activation. One of the mechanisms that Tregs employ to interfere with costimulation is via CTLA4 (or CD152) expression. CTLA4 competes against CD28 to bind to CD80/CD86 on DCs [120,121] and, in this way, prevents the proliferation, activation, and differentiation of Teff cells via CD28. The binding of CTLA4 to CD80/CD86 will also lead to the production of the enzyme indoleamine 2,3 dioxygenase (IDO) by DCs, which initiates the breaking down of the essential amino acid tryptophan into kynurenine. The scarcity of tryptophan suppresses the protein synthesis of Teff cells, resulting in cell cycle arrest and, thus, the inactivity or anergy of Teff cells [122,123,124,125].

Tregs also use the Lag-3 (or CD223) molecule to interact with APCs by binding with the HLA class II molecules with a high affinity, like CD4. Although this does not interfere with the interaction between HLA class II and CD4 on T cells [86], it does have functional consequences for T cell activation, since the binding of Lag-3 to HLA class II suppresses DC maturation and the capacity of DCs to present antigens. Namely, Lag-3 binding induces the cross-linking of HLA class II molecules, and the activation of immunoreceptor tyrosine-based activation motif (ITAM)-mediated inhibitory signaling in DCs via extracellular-signal-regulated kinase (ERK) and protein tyrosine phosphatase 1 (SHP1) [126,127]. 

Additionally, Tregs can express a molecule named TIGIT (T cell immunoreceptor with immunoglobulin and ITIM domain). TIGIT competes with CD226 for its ligand CD155 on DCs. CD226 is a costimulatory receptor that promotes cell contact and TCR signaling [128] and induces the production of proinflammatory cytokines by Teff cells [129]. TIGIT binds to CD155 with greater affinity than CD226, thereby limiting CD226-mediated activation and Teff function [130,131,132]. Additionally, the binding of TIGIT to CD155 on DCs induces CD155 phosphorylation and triggers a signaling cascade, resulting in the increased production of IL-10 and the reduced production of IL-12 by DCs, further inhibiting the activation of Teff cells [131,133]. 

Another important feature of some Tregs is their capacity to modulate proinflammatory DCs to induce new Tregs (Figure 2) [117] and, through this ‘infectious tolerance’, create a legacy of antigen-specific immune regulation that lasts beyond their lifetime. This process requires Treg-mDC contact and the activation of the Tregs by their cognate (auto)antigen, and critically involves immune regulatory molecules such as ICOS-L and B7-H3. Indeed, blocking these molecules prevented the transfer of tolerogenic properties to mature DCs by Tregs [117].

### 4.2. Inhibitory Cytokines

Tregs can suppress immune cells via the release of soluble mediators or inhibitory cytokines such as IL-10, TGF-β, and IL-35 (Figure 2) [134,135]. These cytokines restrict the stimulation and/or survival of Teff cells and induce survival signals in Tregs, supporting peripheral homeostasis [136]. IL-10 inhibits the CD28 pathway, which T cells need as co-stimulation to become activated [137,138]. The active form of TGF-β can function in either a surface-bound or secreted form, after which it binds its receptor on Teff cells [139,140]. This eventually affects the expression of genes such as GATA3, T-bet, STAT4, IFN-γ, and granzyme B, which are important for T cell differentiation and function [141,142,143,144,145]. IL-35 is a heterodimeric cytokine that belongs to the IL-12 family [135] and plays a role in immunosuppression by inhibiting T cell proliferation and promoting the induction of Tregs cells from naïve T cells without the requirement of IL-10, TGF-β, and FOXP3 [146].

### 4.3. Metabolic Disruption

Tregs influence Teff metabolism by competing for IL-2 and by energy deprivation using CD39 and CD73 (Figure 2). Tregs express high levels of CD25, the high-affinity subunit of the IL-2 receptor, thereby possessing a stronger capacity to bind IL-2 than other T cells [122,147]. By limiting IL-2 access for proliferating cells, Tregs trigger metabolic disruption and apoptosis [148,149]. The ATP apyrase (CD39) and ecto-5′-AMP-nucleotidase (CD73) suppress Teff cell function by converting ATP into adenosine [150]. The CD39 expressed by Tregs breaks down ATP into AMP, while CD73 converts AMP into adenosine. When adenosine binds the adenosine receptor (A2A) on Teff cells, this increases c-AMP via the G-protein-coupled receptor and results in an inhibitory signal in T cells [151]. CD39 also functions to sustain Treg stability since CD39^high^ Tregs remain unchanged when cultured in the presence of the proinflammatory cytokines IL-1β and IL-6, whereas CD39^low^ Tregs can differentiate into Th1 and Th17 cells [152]. Also, CD39^high^ Tregs showed stronger suppressive capacity to inhibit PBMCs compared to CD39^low^ Tregs in vitro and protected against xenograft-versus-host disease in a mouse model [152]. Tregs can also disrupt metabolism indirectly by CTLA-4-induced IDO expression in DCs (as described in Section 4.1). IDO breaks down tryptophan and the resulting scarcity of tryptophan suppresses Teff cells [122,123,124,125].

### 4.4. Direct Interaction with Effector T Cells

Tregs can directly interact with Teff cells through the programmed cell death protein (PD-1) and programmed death-ligand 1 (PD-L1) (Figure 2) [153,154]. PD-L1 ligation on CD4 T cells can prevent their activation and polarization [155]. PD-1^high^ Tregs have a much stronger suppressive capacity than PD-1^low^ Tregs during chronic LCMV infection [156]. A PD-1 blockade on PD-1^high^ Tregs prevented the suppression of Teff cells in vitro (stimulated with anti-CD3/CD28 without the presence of APCs), pointing to the direct interaction of PD-1 on Tregs with PD-L1 on Teff. In addition, PD-L1 engagement on memory T cells (CD4^pos^CD25^neg^CD45RA^neg^CD45RO^pos^) promoted their conversion into iTregs when using an antibody crosslinking PD-L1 [157]. It is, therefore, possible that PD-1 on Tregs binds to PD-L1 on Teff, leading to the crosslinking of PD-L1 and the conversion of Teff into iTregs. Furthermore, PD-1 signaling is important for FOXP3 expression and for maintaining Treg homeostasis [158,159].

### 4.5. Induction of Apoptosis

Finally, Tregs can induce apoptosis in Teff cells (Figure 2) through the release of granzyme B (GzmB) and perforin (PFN) [160,161] or by the expression of FAS-L [162,163,164,165]. Tregs can also use β-galactoside binding proteins (galectin) to suppress immune cells. The blockade of galectin-1 (Gal-1) reduced the suppressive capacity of human nTregs in vitro [166]. Galectin 9 (Gal-9) is expressed on the surface of Tregs and binds T cell immunoglobulin and mucin domain 3 (TIM3) on Teff cells, which results in anergy or apoptosis, mediated by intracellular calcium release [167,168]. Furthermore, activated Tregs upregulate the tumor necrosis factor-related apoptosis-inducing ligand (TRAIL), which binds death receptor (DR) 4 and 5 on Teff cells and thereby triggers the intracellular signaling components forming the death-inducing signaling complex (DISC), subsequent caspase cascade activation, and cell death [169].

### 4.6. Evolving Understanding of a Regulatory Role in HLA-DR Expression and Extracellular Vesicles

There are a few additional but so far poorly investigated mechanisms that Tregs may use to suppress immune responses. 

Activated human Teffs and Tregs express HLA class II [170,171,172,173,174], the role of which remains unclear, given that these cells do not have antigen-processing machinery. The antibody blockade of HLA class II resulted in the loss of the suppressive activity of Treg in vitro [170,175]. Tregs could express HLA class II, loaded with self-peptides, to connect with autoreactive Teff cells and use this interaction to inhibit Teff cells via one or several of the regulatory mechanisms described in this review. Alternatively, the expression of HLA class II could allow a self-regulating mechanism through an interaction with Lag-3. 

Another interesting option involves extracellular vesicles (EVs) being released by Tregs. EVs are membranous structures that can be produced by any cell, the content of which reflects the cell of origin. Human Treg EVs can contain immunomodulatory molecules such as CD25, CD73, Nrp1, and CTLA-4 [176], cytokines such as IL-35 [177], and microRNAs such as Let-7d, Let-7b, miR-155, and miR-146a-5p [178,179]. These EVs have been demonstrated to influence processes like miRNA-induced gene silencing, surface protein activity, and enzyme activity in vitro [176,180,181]. Still, Treg EVs were not as effective as Tregs in suppressing Teff, indicating that cell-based processes are indispensable for optimal suppression [178]. Though less effective, EVs from Tregs could contribute to the intercellular exchange with Teff or between Tregs themselves, governing immunological responses and, therefore, establishing a tolerogenic milieu in a cell-free manner. 

The current results raise new questions about the involvement of HLA-DR on Tregs and the release of EVs in the suppressive capacity of Tregs, and both need to be studied more closely to understand their role. 

## 5. A New View on the Treg Universe

Recent advances in multidimensional analyses have enabled us to appreciate the complexity of the Treg universe and that a single marker or a functional property does not suffice to define this cell type. Our unique Treg clones illustrate that even when Tregs share their antigen-specificity and surface phenotypes, they can differ in the number and type of regulatory functions that they use for suppression (Figure 2). As an analogy, some Tregs may have a single inhibitory function in vivo (e.g., the killing of Teffs or APCs), while others may exert multiple immunoregulatory functionalities, irrespective of their origin or antigen specificity. Classifying Tregs based on their different functional properties will help to define, measure, and understand Tregs more accurately, particularly in combination with analyses of the phenotypes that accompany them, to identify or confirm markers that are tightly linked with a certain function, for example, the expression of FAS-L and the production of granzymes associated with killing capacity, and the expression of CTLA4 with the conditioning of APCs. Such markers can be used to assign the functional property of a certain Treg and allow us to dissect the regulatory T cell population in general, instead of focusing on ‘specific’ Treg subtype definitions such as Tr1 and Tr2 cells (defined on the basis of different markers in different studies). 

In conclusion, we propose to view the Treg universe with fresh eyes and consider the functional properties of Tregs and the related markers, rather than merely the expression of FOXP3 or any other supposedly Treg-specific markers. In this way, we hope that a consensus can be reached on characterizing human Tregs and studying Tregs categorically. This will also help to relate Tregs induced in vitro with Tregs ex vivo, which is difficult at this moment.

However, there are reassuring examples that islet-antigen-specific Tregs that are induced in vitro by tolDC are very similar to Tregs that are isolated and expanded ex vivo [49,111]. Furthermore, identifying Tregs by function may help in moving forward strategies that aim to employ Tregs in immunotherapy. It is conceivable that certain Treg entities are superior to others, which may explain and overcome the disappointing results from various Treg-based immune intervention trials (as reviewed in [182]).

## Figures and Tables

**Table 2:** Summary data of Treg clusters induced by tolerogenic dendritic cells (tolDCs) against proinsulin peptide.

	Cluster 1	Cluster 2	Cluster 3
**Mechanism of priming**	iTregs primed by tolDCs	iTregs primed by tolDCs	iTregs primed by tolDCs
**Function**	Monocyte killing Inhibition of naïve T cells and Th1 responses	Monocyte killing	Monocyte killing Strong inhibition of naïve T cells and Th1 responses
**Expression of markers**	High amounts of IL-10, TNF, IFN-γ, and IL-13 CTLA4 Granzyme B	Granzyme B	CTLA4 Some make IL-10
**Peculiarity**	Reversed TCR docking		Infectious tolerance

## Abbreviations

**APCs**	Antigen-presenting cells
**CTLA4**	Cytotoxic T-lymphocyte-associated protein 4
**DR**	Death receptor
**EVs**	Extracellular vesicles
**Gal**	Galectin
**GITR**	Glucocorticoid-induced TNFR-related protein
**GPA33**	Glycoprotein A33
**GzmB**	Granzyme B
**IDO**	Indoleamine 2,3 dioxygenase
**IPEX**	Immune dysfunction/polyendocrinopathy/enteropathy/X-linked
**iTregs**	Tregs induced in vitro
**Lag-3**	Lymphocyte-activation gene 3
**LAP**	Latency-associated peptide
**MAGE-A3**	Melanoma antigen family A3
**mDCs**	Inflammatory monocyte-derived dendritic cell
**Nrp-1**	Neuropilin
**nTregs**	Naturally occurring Tregs
**PD-1**	Programmed cell death protein
**PD-L1**	Programmed death-ligand 1
**PFN**	Perforin
**pTregs**	Tregs generated in the peripheral tissues
**TCR**	T cell receptor
**Teff**	Effector T cells
**TIGIT**	T cell immunoreceptor with immunoglobulin and ITIM domain
**TIM3**	T cell immunoglobulin and mucin domain 3
**tolDCs**	Tolerogenic dendritic cells
**Tr1**	Type 1 regulatory T cells
**Tr2**	Type 2 regulatory T cells
**TRAIL**	Tumor necrosis factor-related apoptosis-inducing ligand
**Tregs**	Regulatory T cells
**TSDR**	Treg-specific demethylated region
**tTregs**	Tregs generated in the thymus
